# Compound Danshen Dripping Pill inhibits high altitude-induced hypoxic damage by suppressing oxidative stress and inflammatory responses

**DOI:** 10.1080/13880209.2021.1998139

**Published:** 2021-11-22

**Authors:** Yunhui Hu, Jia Sun, Tongxing Wang, Hairong Wang, Chunlai Zhao, Wenjia Wang, Kaijing Yan, Xijun Yan, He Sun

**Affiliations:** a GeneNet Pharmaceuticals Co. Ltd, Tianjin, P.R. China; b The State Key Laboratory of Core Technology in Innovative Chinese Medicine, Tasly Academy, Tasly Holding Group Co., Ltd, Tianjin, China; c Tasly Pharmaceutical Group Co., Ltd, Tianjin, China

**Keywords:** Hypobaric hypoxia, traditional Chinese medicine, NF-κB, Nrf2, inflammation

## Abstract

**Context:**

Previous studies indicate that compound Danshen Dripping Pill (CDDP) improves the adaptation to high-altitude exposure. However, its mechanism of action is not clear.

**Objective:**

To explore the protective effect of CDDP on hypobaric hypoxia (HH) and its possible mechanism.

**Materials and methods:**

A meta-analysis of 1051 human volunteers was performed to evaluate the effectiveness of CDDP at high altitudes. Male Sprague-Dawley rats were randomized into 5 groups (*n* = 6): control at normal pressure, model, CDDP-170 mg/kg, CDDP-340 mg/kg and acetazolamide groups. HH was simulated at an altitude of 5500 m for 24 h. Animal blood was collected for arterial blood-gas analysis and cytokines detection and their organs were harvested for pathological examination. Expression levels of AQP1, NF-κB and Nrf2 were determined by immunohistochemical staining.

**Results:**

The meta-analysis data indicated that the ratio between the combined RR of the total effective rate and the 95% CI was 0.23 (0.06, 0.91), the SMD and 95% CI of SO_2_ was 0.37 (0.12, 0.62). Pre-treatment of CDDP protected rats from HH-induced pulmonary edoema and heart injury, left-shifted oxygen-dissociation curve and decreased P50 (30.25 ± 3.72 vs. 37.23 ± 4.30). Mechanistically, CDDP alleviated HH-reinforced ROS by improving SOD and GPX1 while inhibiting pro-inflammatory cytokines and NF-κB expression. CDDP also decreased HH-evoked D-dimer, erythrocyte aggregation and blood hemorheology, promoting AQP1 and Nrf2 expression.

**Discussion and conclusions:**

Pre-treatment with CDDP could prevent HH-induced tissue damage, oxidative stress and inflammatory response. Suppressed NF-κB and up-regulated Nrf2 might play significant roles in the mechanism of CDDP.

## Introduction

Acute high-altitude hypoxia affects the blood flow and the efficiency of oxygen utilization, causing multi-organ injury and ultimately leading to life-threatening high-altitude cerebral edoema (HACE) or high-altitude pulmonary edoema (HAPE) (Imray et al. 2010). Decreased barometric pressure and subsequent reduction in available oxygen are the primary causal factors in these medical conditions (Clarke 2006). Acetazolamide, dexamethasone and montelukast are widely used to prevent acute altitude sickness. However, they produce a variety of adverse effects, including headache, sensory abnormalities, cardiopalmus osteoporosis and increased risk of infection (Fagenholz et al. 2007; Nieto Estrada et al. 2017). Thus, there is an increasing need for the development of alternative therapies to treat and prevent these conditions.

Hypoxia rapidly increases the levels of free radicals in cells and whole-body hypoxia overwhelms the reserves of scavengers, leading to the accumulation of reactive oxygen species (ROS), the release of inflammatory factors and injury of red blood cells (RBCs), vascular endothelial cells (VECs) and other tissues, such as lung, brain and heart (Lisk et al. 2013). Therefore, it is important to identify effective antioxidant and anti-inflammatory drugs for the prevention of hypoxia-related disorders. Some studies indicate that antioxidant supplementation diminishes acute mountain sickness (AMS) incidence (Bailey and Davies 2001; Fan et al. 2013; Zhang et al. 2020), suggesting that antioxidants could be used as a protective measure against acute hypoxia injury.

Compound Danshen Dripping Pill (CDDP, T89, Dantonic^®^), which is a traditional Chinese medicine produced by combining traditional Chinese and modern medical technologies, contains *Saviae miltiorrhizae* Bunge (Lamiaceae), *Panax notoginseng* Burkill (Araliaceae) and borneol (Guo et al. 2016). Many studies have indicated the antioxidant and anti-inflammatory properties of CDDP in the cardiovascular system, and it has been widely used for the prevention and treatment of acute myocardial ischaemia and other cardiovascular diseases for over 25 years (Yao et al. 2015; Liang et al. 2018; Yao et al. 2019). Clinical studies indicate that CDDP can also improve oxygen saturation (SO_2_) and prevent or relieve AMS-related symptoms as well as hypoxia-induced tissue damage (Li, Li, et al. 2020; Li, Guo, et al. 2020). However, other CDDP protective mechanisms under acute high-altitude hypoxia remain elusive.

In the present study, we performed a meta-analysis of clinical studies in order to evaluate the efficacy of CDDP in maintaining SO_2_ and relieving high-altitude hypoxia-related symptoms. Furthermore, using a rat model of high-altitude hypoxia we demonstrate that pre-treatment with CDDP prevents hypobaric hypoxia-induced tissue damage, oxidative stress and inflammatory response and offer data suggesting that this effect is due to suppression of NF-κB and up-regulation of AQP1 and Nrf2.

## Materials and methods

### Meta-analysis of randomized controlled trials

Data from randomized controlled trials reporting the use of CDDP for prevention or treatment of hypobaric hypoxia were obtained from China National Knowledge Infrastructure (CNKI) and PubMed databases. The search terms were ‘Danshen Dropping Pills’, ‘Compound Danshen Dripping Pills’, ‘Fufang Danshen Diwan’, ‘T89’, ‘Dantonic’ and ‘Cardiotonic Pills’. As of April 30, 2021, six studies (Zhang et al. 2008; Tian et al. 2012; Liu and Zhang 2017; Feng 2019; Li, Li, et al. 2020; Li, Guo, et al. 2020) involving 1051 patients were obtained.

Two reviewers independently, and in duplicate, evaluated the risk of bias of the eligible studies according to the assessment tool of the Cochrane Handbook for Systematic Reviews of Interventions 4.2.2 (Higgins and Green 2011). The criteria included: (1) The sequence generation; (2) The allocation concealment; (3) The blinding of participants and personnel; (4) The blinding of outcome assessments; (5) Incomplete outcome data; (6) Selective reporting; and (7) Other sources of bias. A meta-analysis was conducted using Review Manager 5.3 (Cochrane; www.cochrane.org/). The risk ratio (RR) with a 95% CI was used for the improvement of symptoms, while the standardized mean difference (SMD) with a 95% CI was adopted for the change of SO_2_.

### Chemicals and reagents

CDDP was obtained from Tasly Pharmaceutical Co., Ltd. (Tianjin, China). Acetazolamide (ACTZ) was purchased from Shanghai Yuanye Biotechnology (Shanghai, China). Rabbit anti-AQP1 (A15030), NF-κB (A11201) and Nrf2 (A0674) polyclonal antibodies were purchased from ABclonal Biotechnology Co., Ltd (Wuhan, Hubei, China).

### Animals, treatments and sample preparation

Male Sprague-Dawley rats were obtained from SBF Biotechnology (Beijing, China). Rats were distributed randomly in 5 groups (6 rats per group) and received treatment by intragastric administration at 10 mL/kg once a day for 3 d. Treatments consist of 0.5% sodium carboxymethylcellulose (CMC), CDDP (at 170 and 340 mg/kg, in 0.5% CMC) and 50 mg/kg ACTZ in 0.5% CMC. Rats in the control group (0.5% CMC) were left at atmospheric pressure, whereas animals in all other treatments, including a model group treated with only 0.5% CMC, were introduced in a hypobaric chamber (QTK-LP; IVD Biotechnology, Henan, China) for 24 h in order to simulate an altitude of 5500 m.

**Figure 1.**
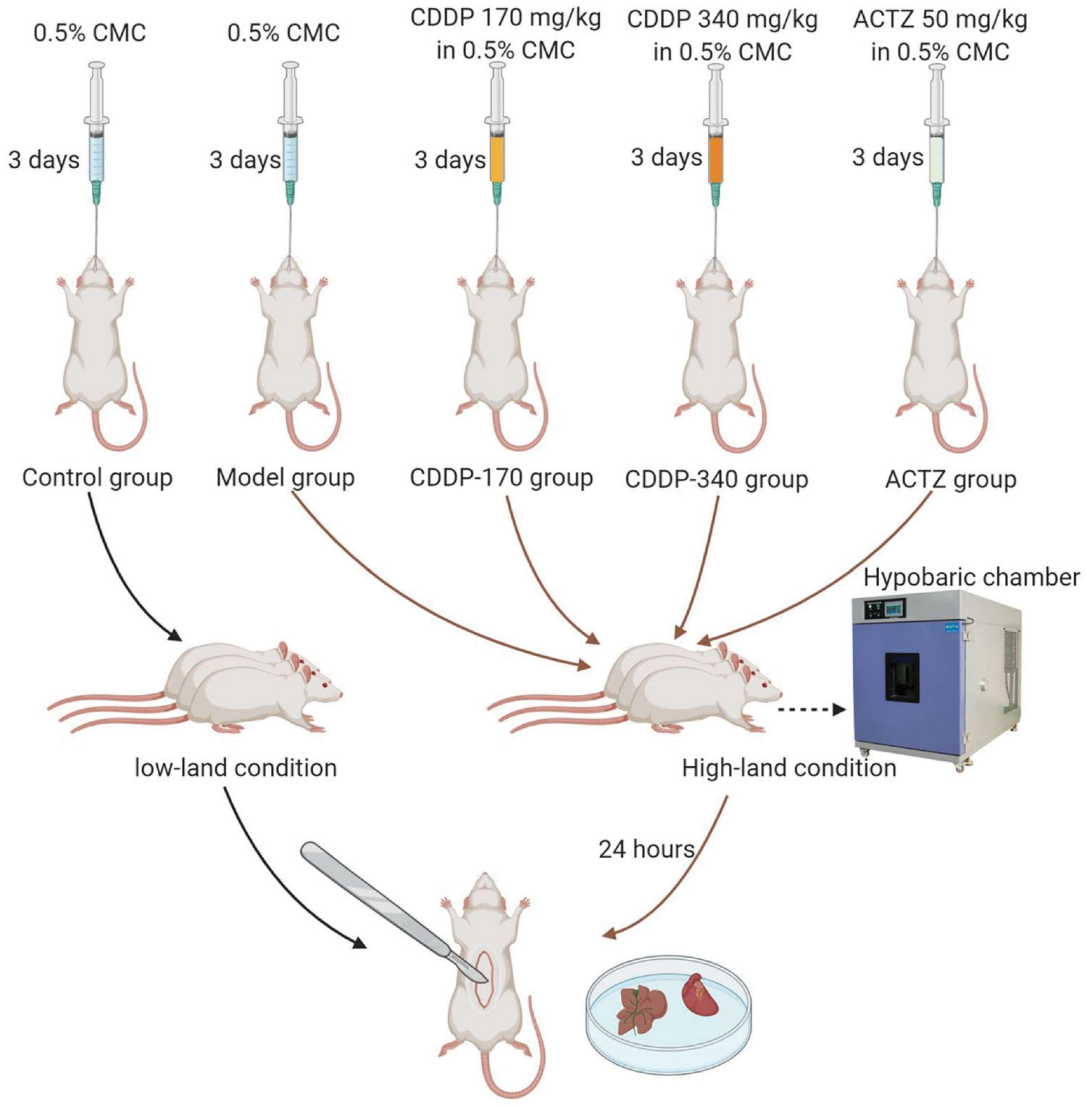
Workflow for a hypobaric hypoxic model establishment and experimental design of treatment for each group.

Rats were then anesthetized, blood collected from the abdominal aorta, sacrificed and their organs harvested. A summary of the experimental strategy is shown in Figure 1. Animals were housed under specific pathogen-free conditions with free access to water and food at the animal centre of Tianjin Pharmaceutical Research Institute in China. All animal experiments complied with the Guidance of the protocol IACUC-2020042603 approved by the Institutional Animal Care and Use Committee (IACUC) of Tianjin Pharmaceutical Research Institute in China.

### Arterial blood-gas analysis and fitting of the oxygen-dissociation curve

Rat arterial blood (1 mL) was used for blood-gas analyses. The partial pressure of oxygen (PO_2_), SO_2_, and pH were detected at 37 °C by microelectrodes in a blood gas analyzer (iSTAT-300; Abbott Laboratories, Chicago, IL, USA). The oxygen-dissociation curve and the pressure of oxygen when SO_2_ level reaches 50% (P50) was calculated according to the Hill equation (Balaban et al. 2013):

(a) $${\mathrm{SO}}_{2}=K\times {{\mathrm{PO}}_{2}}^{n}/(1+K\times {\mathrm{PO}}_{2})$$

(b) $$K\times {{\mathrm{PO}}_{2}}^{n}={\mathrm{SO}}_{2}/(100–{\mathrm{SO}}_{2})$$

(c) $${\mathrm{Log}}_{10}\;{\mathrm{SO}}_{2}/(100–{\mathrm{SO}}_{2})=\log_{10}\;K+n\;\log_{10}{\mathrm{PO}}_{2}$$

In Equation (a), *K* is the oxygen dissociation constant, and *n* is the Hill coefficient. We converted Equation (a) into Equation (b) mathematically. After taking the logarithm on both sides of Equation (b), we obtained Equation (c) for a linear equation in the form of *Y* = a*X* + b. When we made *Y* = log_10_ SO_2_/(100 – SO_2_) and *Y* = log_10_ PO_2_, the slope ‘a’ became the ‘*n*’ value of the Hill equation, and b the logarithmic value of *k*. We calculated *n* and *K* values using the data for PO_2_ and SO_2_ of each group. After the regression, and making PO_2_ range from 0 to 100, the oxygen-dissociation curve was fitted and P50 was calculated.

### Measurement of antioxidant status and inflammatory cytokines

To evaluate the antioxidant status, levels of ROS, TAOC, SOD and GPX1 were measured using commercial assay kits. Rat heart and lung tissues were weighed and converted to a cell suspension using a 300-mesh sieve. From each rat, whole blood (1 mL) was taken for a ROS test along with heart cells/lung cells following the manufacturer’s instructions of a ROS assay kit (E004-1-1; Jiancheng Bioengineering Institute, Nanjing, China). Plasma was obtained by removing blood cells through centrifugation at 1800 *g* for 15 min at room temperature. TAOC was measured according to the instructions of a TAOC Assay Kit (A015; Jiancheng Bioengineering Institute, Nanjing, China). The concentrations of SOD, GPX1, NT-proBNP and inflammatory cytokines IL-1, IL-6, TNF-α, ICAM1, MMP9 were determined using commercial ELISA kits (Jiancheng Bioengineering Institute, Nanjing, China) according to the manufacturer’s instructions (Supplementary Table S1), Samples from every rat were detected separately.

### Red blood cell (RBC) aggregation index and hemorheology

Rheology tests were undertaken by using blood specimens to ascertain the viscosity of whole blood under a ‘high’ (1/200), ‘medium’ (1/60) and ‘low’ (1/10) shear rate using an automatic blood rheometer (LBY-6COMPACT, Precil Instrument Co., Ltd, Beijing, China). The RBC aggregation index was calculated from the ratio of low shear to high shear viscosities. The concentration of D-Dimer was determined using a commercial assay kit according to the manufacturer’s instruction (E029-1-1; Jiancheng Bioengineering Institute, Nanjing, China).

### Haematoxylin and eosin (H&E) staining and immunohistochemical (IHC) detection

Rat heart and lung tissues were fixed in formalin and stained with H&E using standard histological procedures. Expression of AQP1, NF-κB and Nrf2 in these two organs was detected by standard IHC staining essentially as described (Hu et al. 2018). Three randomly selected fields were photographed with a Nikon Eclipse Ti-SR microscope (Nikon, Japan).

### Statistical analyses

Values are expressed as mean ± SEM. All experiments were repeated at least 3 times independently. Initially, all data were conducted the normal distribution analysis with GraphPad Prism. The data followed normal distribution were then analyzed by a one-way ANOVA with Tukey post-test by SPSS software (SPSS Inc., Chicago, IL, USA). Significant difference was considered if *p*-value < 0.05 (*n* ≥ 3).

## Results

### CDDP attenuates hypobaric hypoxia symptoms and increases SO_2_

**Figure 2.**
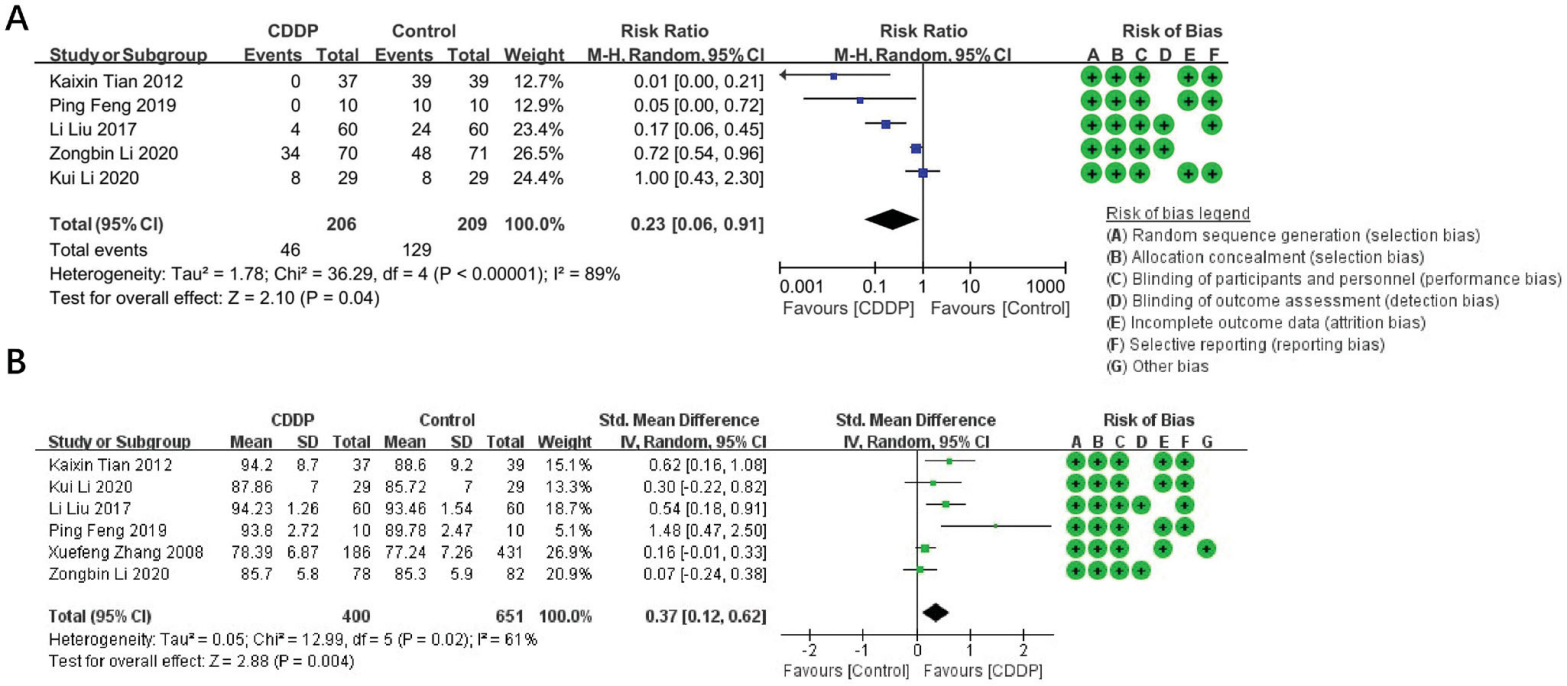
Effect of CDDP on the alleviation of hypobaric hypoxia symptoms (A) and improvement of SO_2_ (B) in people exposed to hypobaric hypoxia and summary of bias risks: the reviewers’ judgement of risk for each bias item in each included study.

Five trials were selected for the evaluation of CDDP effectiveness in alleviating hypobaric hypoxia symptoms. The meta-analysis indicated that the ratio between the combined RR of the total effective rate and the 95% CI was 0.23 (0.06, 0.91) (Figure 2(A)), suggesting that CDDP can significantly alleviate the symptoms of hypobaric hypoxia.

The changes of SO_2_ due to CDDP treatment were reported in six trials. The meta-analysis indicated that the SMD and 95% CI of SO_2_ was 0.37 (0.12, 0.62) (Figure 2(B)), suggesting that CDDP can significantly improve SO_2_.

### CDDP alleviates tissue damage and improves tissue oxygenation in rats exposed to acute hypobaric hypoxia

**Figure 3.**
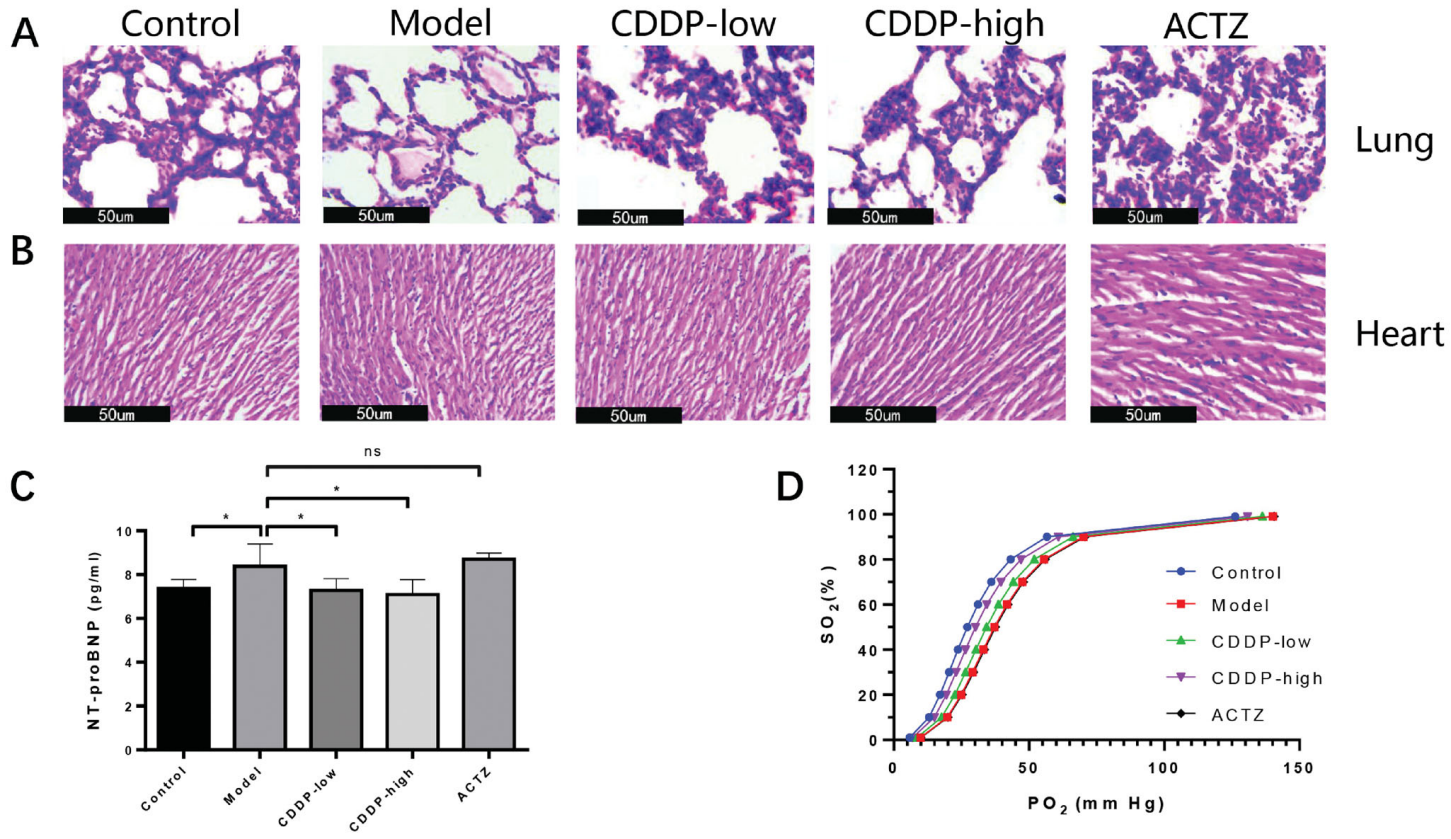
CDDP alleviates hypobaric hypoxia-induced tissue damage and improves tissue oxygenation. HE staining of rat lung (A) and heart (B) sections. Representative pictures from three randomly selected fields are shown. Black bar represents 50 μm. (C) Expression level of blood NT-proBNP. Statistical comparisons were made against the model group. Data indicate the average ± SEM of at least three independent experiments. *p < 0.05. (D) Oxygen dissociation curve determined by arterial blood gas analysis and calculated according to the Hill equation. Arterial blood gas values and oxygen-dissociation curve parameters are fully described in Table 1.

In order to investigate the function of CDDP in tissue damage caused by hypobaric hypoxia, we used a hypobaric chamber to simulate high altitude low pressure. Exposure to hypobaric hypoxia for 24 h induced pulmonary edoema and vacuoles, which were alleviated by CDDP or ACTZ (Figure 3(A)). ACTZ is a drug approved by US Food and Drug Administration for AMS and used in our study as a reference control. Although no obvious damage could be observed in H & E staining of heart tissue (Figure 3(B)), expression of N-terminal pro-brain natriuretic peptide (NT-proBNP), an important marker indicating injured myocardium (Hall 2004), increased in the rats exposed to hypobaric hypoxia. However, CDDP administration, but not ACTZ, attenuated its increase significantly (Figure 3(C)).

Oxygen tension at 50% haemoglobin saturation (P50), which reflects the degree of peripheral oxygen offloading and tissue oxygenation, is a parameter that indicates the inverse effect of the affinity between haemoglobin and oxygen (Mairbäurl and Weber 2012) and is used to measure the development of hypobaric hypoxia symptoms. We observed that hypobaric hypoxia significantly increased P50 values and left-shifted the oxygen-dissociation curve in rats, which were reversed by CDDP in a dose-dependent manner (Figure 3(D) and Table 1), suggesting CDDP facilitates the improvement of tissue oxygenation. Importantly, the ACTZ administration didn’t improve P50.

**Table 1.** Arterial blood-gas values and oxygen-dissociation curve parameters.

Experiment groups	PO_2_	SO_2_	*n*	logK	P50
Control	87.67 ± 4.84	97.00 ± 0.63*	2.99 ± 0.15**	4.29 ± 0.37**	27.19 ± 3.16**
Model	73.33 ± 15.91	89.83 ± 5.91	3.479 ± 0.20	–5.45 ± 0.49	37.23 ± 4.30
CDDP-low	90.83 ± 12.07	95.33 ± 3.78	3.33 ± 0.25	–5.11 ± 0.62	34.28 ± 5.51
CDDP-high	93.67 ± 6.80*	97.00 ± 0.89*	3.13 ± 0.18**	4.65 ± 0.43**	30.25 ± 3.72**
ACTZ	68.67 ± 33.13	80.33 ± 18.73	3.37 ± 0.15	–5.20 ± 0.35	35.08 ± 3.10

All statistical comparisons were made against the model group. *p < 0.05, **p < 0.01

Thus, these data indicate that CDDP alleviates tissue damage and improves tissue oxygenation in rats exposed to acute hypobaric hypoxia.

### CDDP antagonizes oxidative stress in rats during hypobaric hypoxia

**Figure 4.**
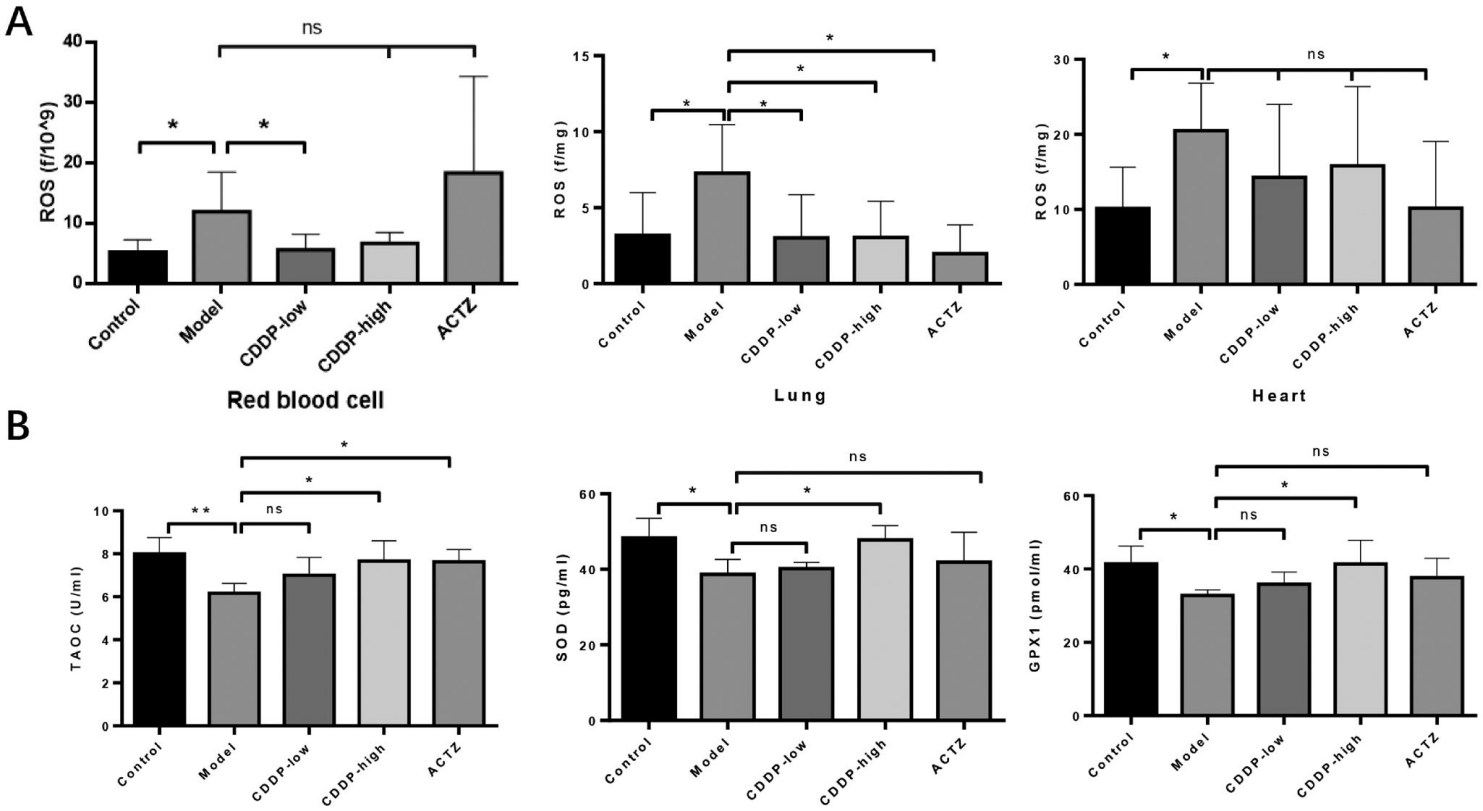
CDDP attenuates hypobaric hypoxia-induced oxidative stress. Blood, lung and heart samples collected from rats have conducted the following assays. (A) ROS levels determined by the assay kit. (B) Serum TAOC, SOD and GPX1 levels detected by the assay kit. Samples from every rat were detected separately. Data indicate the average ± SEM of at least three independent experiments. *n* = 6 of each group. *p < 0.05, **p < 0.01.

In order to elucidate the mechanism underlying CDDP-mediated protective role in hypobaric hypoxic tissue damage, we explored the effects of CDDP on oxidative stress, which has been confirmed as responsible for the development of high altitude-triggered tissue damage (Dosek et al. 2007). Exposure to hypobaric hypoxia triggered the production of ROS relatively to the control group, which was inhibited following CDDP treatment in lung and red blood cells (high dose group), but not the heart. ACTZ treatment did not inhibit ROS formation in RBCs or the heart but did so in the lung (Figure 4(A)). We further investigated the effect of CDDP treatment on the antioxidant status measured in plasma. ACTZ restored TAOC, but not SOD or GPX1 levels, but high dose CDDP treatment restored the levels of the three markers (Figure 4(B)). Thus, CDDP inhibits oxidative stress in rats during hypobaric hypoxia.

### Treatment with CDDP blunts hypobaric hypoxia-evoked inflammatory response and thromboembolic risk in rats

**Figure 5.**
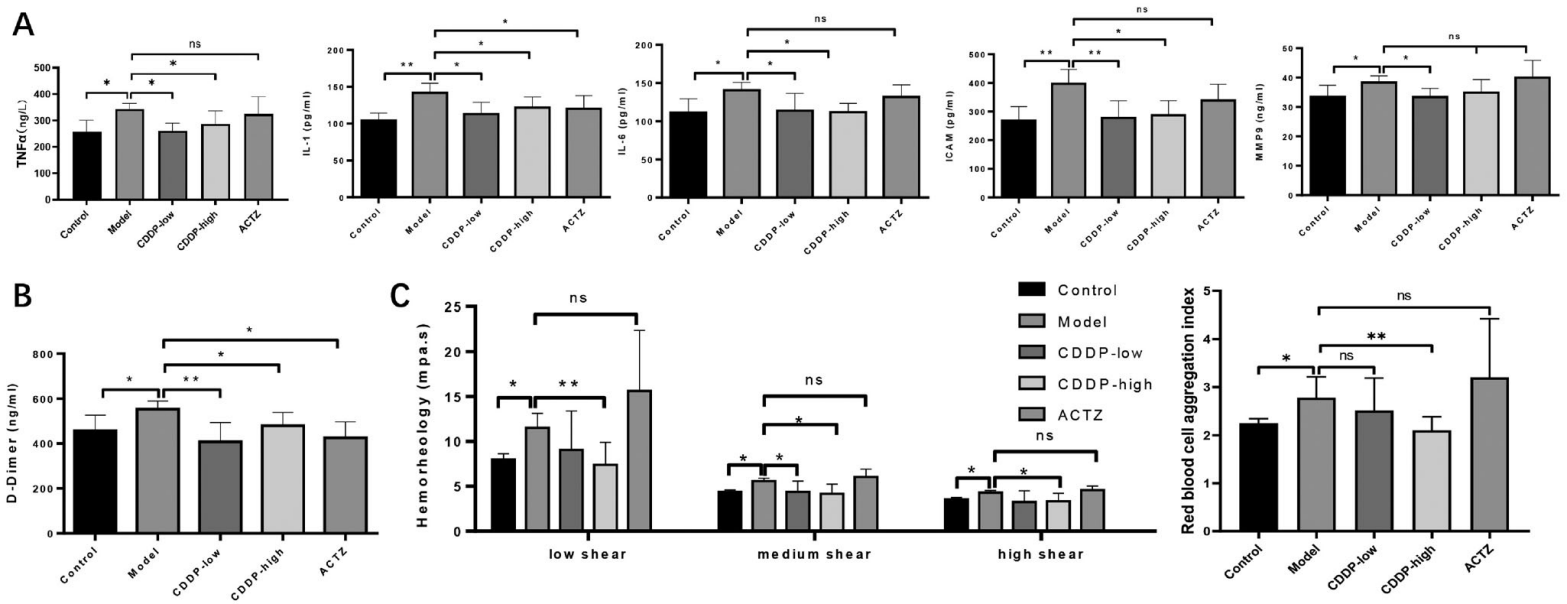
CDDP alleviates inflammation and blood viscosity in hypobaric hypoxia-stimulated rats. Determination of inflammatory cytokines concentration by ELISA (A) and D-Dimer levels determined by assay kit (B). (C) Blood hemorheology determined using a blood rheometer (left panel); Red blood cell aggregation index was calculated according to blood hemorheology (right panel). Samples from every rat were detected separately. Data indicate the average ± SEM of at least three independent experiments. *n* = 6 of each group. *p < 0.05, **p < 0.01.

In order to elucidate the mechanisms by which CDDP protects rats against hypobaric hypoxia, we explored CDDP effects on the inflammatory response. Inflammatory response, with increased inflammatory cytokine levels, is a critical cause of tissue damage under various adverse conditions including hypobaric hypoxia exposure (Wang et al. 2018). Increased TNF-α serum levels were detected in hypobaric hypoxia-exposed rats, but this elevation was blocked by CDDP, although not by ACTZ, pre-treatment (Figure 5(A)). CDDP also suppressed the increase in pro-inflammatory cytokines IL-1, IL-6, ICAM1 and MMP9 in rat serum upon hypobaric hypoxia condition. Importantly, ACTZ only suppressed IL-1 increase, but not IL-6, ICAM1 or MMP9 (Figure 5(A)). This indicates that CDDP inhibits the inflammatory response.

Some studies show evidence of thrombosis and procoagulatory activity under hypobaric hypoxia (Mannucci et al. 2002; Torgovicky et al. 2005). Elevated D-dimer, RBCs aggregation and rheology parameters have been demonstrated to be involved in thrombosis in various disease models including hypobaric hypoxia exposure (Barshtein et al. 2007; Guilbert et al. 2017). In order to explore the effect of CDDP pre-treatment on these parameters, we measured D-dimer concentration, erythrocyte aggregation and blood hemorheology in our rat hypobaric pressure model system. Results indicate that both CDDP and ACTZ restored the elevation of D-dimer levels due to hypobaric hypoxia, but only high dose CDDP pre-treatment reduced the increase in hypobaric hypoxia-triggered RBC aggregation to levels found in the control group maintained at normal pressure (Figure 5(B)). Importantly, CDDP, but not ACTZ, pre-treatment abolished the increase in RCB aggregation due to hypobaric hypoxia (Figure 5(C)). Thus, pre-treatment with CDDP in this rat model of hypobaric hypoxia reduces the risk of thrombosis and severity of hypoxia.

### Identification of CDDP-protected targets against hypobaric hypoxia-induced tissue injury

**Figure 6.**
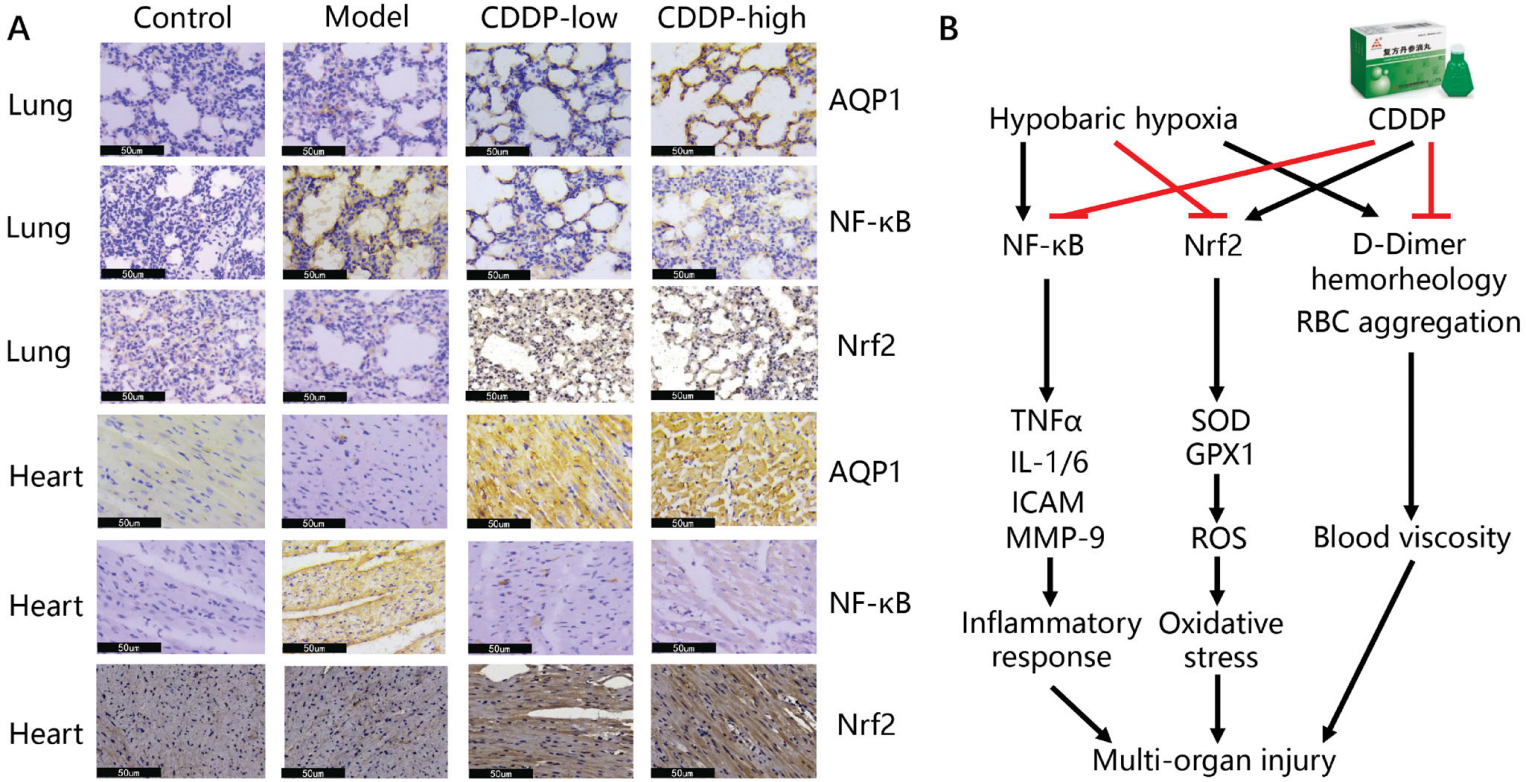
(A) Identification of the CDDP-protected targets against hypobaric hypoxia. Lung and heart samples collected from rats were fixed in formalin, embedded in paraffin, and sectioned for IHC staining using the corresponding antibodies. Representative pictures from three randomly selected fields are shown. Black bar represents 50 μm. (B) Mode of action of CDDP in treating hypobaric hypoxia.

In order to shed some further light on the possible mechanisms for CDDP protection against hypobaric hypoxia in our rat model, we determined the expression of several markers by IHC. AQP1 has a protective role by attenuating tissue edoema and inflammation (Dong et al. 2012; Ding et al. 2013). Nrf2 is a key transcription factor in anti-oxidative stress (Lisk et al. 2013). NF-κB is a heterodimeric protein composed of members of the Rel family of transcription factors and can induce a series of pro-inflammatory cytokines, including TNF-α, IL-1, IL-6 and MMP9 (Hsieh et al. 2014; Pan et al. 2020). Our results show that CDDP pre-treatment increases the levels of AQP1 and Nrf2 in both pulmonary and heart tissues (Figure 6). Importantly, and corroborating the anti-inflammatory action of CDDP demonstrated above (Figure 5(A)), NF- κB levels did not increase due to hypobaric hypoxia in the heart and lungs of rats pre-treated with CDDP (Figure 6). Thus, the anti-inflammatory action of CDDP can be attributed to its NF-κB inhibitory capacity.

## Discussion

Hypobaric hypoxia, which causes insufficient oxygen availability, leads to multi-organ injury and irreversible tissue damage. Mechanistically, this has been attributed to the large number of oxygen radicals produced by hypobaric hypoxia (Dosek et al. 2007). In clinical trials, CDDP has been confirmed to maintain SO_2_ and to decrease the incidence of AMS (Li et al. 2020). In the present study, the beneficial efficacy of CDDP in improving the adaptation to high altitude exposure was further evaluated through meta-analysis and the anti-hypoxic effects of CDDP pre-treatment were confirmed in a rat model of hypobaric hypoxia.

CDDP has been approved by the China Food and Drug Administration for the treatment of coronary heart disease (CHD) for more than 25 years. Numerous studies have demonstrated the therapeutic properties of CDDP through promoting blood circulation, reducing blood viscosity, inhibiting leukocyte adhesion and inflammation, protecting endothelial cells and optimizing myocardial energy metabolism to ameliorate angina symptoms caused by myocardial ischaemia (hypoxia) (Liang et al. 2018; Yao et al. 2019). Oxidative stress and inflammation caused by the imbalance between demand and supply of oxygen are two common pathophysiological processes shared by myocardial ischaemia and hypobaric hypoxia. This suggests that the protective effects of CDDP against acute high-altitude hypoxia and CHD might share common regulatory mechanisms. Our meta-analysis results indicate a beneficial effect of CDDP in alleviating hypobaric hypoxia symptoms. The incidence of hypobaric hypoxia-related syndromes was reduced, whilst SO_2_ increased significantly in people treated with CDDP. As hypoxia is believed to be the main factor in AMS occurrence and development (Roach and Hackett 2001; Li et al. 2018), we propose that SO_2_ recovery is one of the main contributors to reducing acute high-altitude hypoxia. However, the number of studies used for the analysis is relatively small and many suffer from high heterogeneity. In addition, all the studies used Chinese participants and it is thus unclear whether these results can be extrapolated to other ethnicities. Thus, our conclusions should be treated with a certain amount of caution until larger studies are performed.

In the present study, an acute hypobaric hypoxia model was successfully established, including SO_2_ dropping and antioxidant defense system dysfunction. In line with previous clinical studies, pre-treatment with CDDP helped maintain SO_2_/PO_2_, and left-shifted the oxygen-dissociation curve, indicating CDDP increased the oxygen affinity of haemoglobin which would augment arterial SO_2_ during hypoxia. We found that hypobaric hypoxia decreases oxygen affinity, leading to increased ROS accumulation and inflammatory factors generation (Ciaccio et al. 2017; Pajuelo Reguera et al. 2020). Importantly, pre-treatment with CDDP exerted strong antioxidant capacity through promoting TAOC and expressions of SOD and GPX1, two important antioxidant enzymes to scavenge ROS in the whole body (Wei et al. 2001). In addition, CDDP counteracted the enhanced inflammation in acute hypobaric hypoxic rats by muting pro-inflammatory cytokines MMP-9, ICAM1, IL-1, IL-6 and TNFα levels. CDDP administration has been demonstrated to ameliorate inflammation and oxidative stress in several myocardial ischaemia animal models (Wei et al. 2013; Han et al. 2017), diabetic mice (Zheng et al. 2011; Zhou et al. 2015; Lu et al. 2016), as well as in the serum of patients who suffered by acute myocardial infarction (A et al. 2020), suggesting that the antioxidant and anti-inflammation capacities of CDDP open a wide window of therapeutic interventions.

It has been previously reported that coagulation parameters and blood rheology change significantly during hypobaric hypoxia (Reinhart et al. 1991; Toff et al. 2006; Jacqueline et al. 2010), in line with results of epidemiological studies indicating that high altitude poses a risk for developing thrombosis (Yanamandra et al. 2020). Previous research has indicated that *Salvia miltiorrhiza* and pseudo-ginseng (*Panax notoginseng*), the two main ingredients of CDDP, are effective in promoting blood circulation and dispersing stasis. Moreover, pharmacological studies have demonstrated that they reduce blood viscosity, accelerate erythrocyte flow, inhibit platelet adhesion and aggregation, and regulate both internal and external blood coagulation (Han et al. 2017; Li et al. 2018; Jia et al. 2019; Ren et al. 2019). In agreement with this large body of evidence, and using a rat hypobaric hypoxia model system, we find that exposure to hypobaric hypoxia causes elevated D-Dimer, erythrocyte aggregation index and parameters of blood rheology, which decreased to a normal level after CDDP treatment, especially in the high dose group. This suggests that the role of CDDP modulating coagulation parameters and blood rheology is not limited to cardiovascular disease, but operates also in acute high-altitude hypoxia.

Regarding the mechanism of CDDP’s protective role in hypobaric hypoxia, we suggest that inhibition of NF-κB and increase in AQP1 and Nrf2 may play important roles. AQP1 provides the principal route for osmotically driven water transport across the epithelial and endothelial barriers and plays an important role in edoema formation during hypoxia (Li et al. 2015; Tan et al. 2020). In addition, AQP1 reduces pulmonary and myocardial edoema to protect lung and cardiac function (Li et al. 2011; Li et al. 2015). Moreover, dysregulated NF-κB and Nrf2 pathways caused by hypoxia have also been demonstrated to induce oxidative stress and inflammatory response (Tripathi et al. 2019; Xiang et al. 2020). Therefore, inhibiting NF-κB or activating Nrf2 alleviates high altitude-induced tissue damage via suppressing oxidative stress and inflammatory response (Lisk et al. 2013; Gong et al. 2018; Pan et al. 2020). Results from our study using a rat model of hypobaric hypoxia indicate that CDDP exhibits wide synergistic effects against hypobaric hypoxia via multiple targets and highlights the holistic approach of traditional Chinese medicine.

ACTZ is the only drug approved by US Food and Drug Administration for AMS, although it has several side effects such as drowsiness, loss of appetite, nausea, vomiting, and diarrhoea (Collier et al. 2016; Harrison et al. 2016; Ono et al. 2017). In the present study, ACTZ was used as a positive control to compare the effects of CDDP, but ACTZ was not as effective as CDDP. In line with these results, ACTZ neither degrades hypoxia-induced H_2_O_2_, even with 200 mg/kg treatment in rats (Fan et al. 2013) nor activates Nrf2 both *in vitro* or *in vivo* (Lisk et al. 2013). Other studies also reported that oxygen saturation does not change following ACTZ treatment both in rats (Wang et al. 2016) and healthy volunteers (Hackett et al. 1985). It has been indicated that the mechanism by which ACTZ protects from hypoxia may be related to the reduction of several pro-inflammatory cytokines (Wang et al. 2016), which is consistent with our experimental results, suggesting that ACTZ and CDDP mechanisms of action may not be identical. Our data indicate that CDDP has a stronger antioxidant efficacy than ACTZ and offers a strong rationale for promoting CDDP use for people under the harsh environment of high-altitude hypoxia. However, we have used only one animal model and set of conditions not leading to severe pulmonary edoema, brain edoema, myocardial damage or death caused by hypobaric hypoxia. Therefore, further investigations focussing on the protective effects and related mechanisms of CDDP on HAPE, HACE or mortality are required.

## Conclusions

Pre-treatment with CDDP prevents hypobaric hypoxia-induced tissue damage, oxidative stress and inflammatory response in a rat model of high-altitude hypoxia. Suppressed NF-κB and up-regulated Nrf2 may play a role in CDDP mechanisms of action.

## Supplementary Material

Supplemental Material

## References

[CIT0001] A X, Li Z, Luo W, Chai J. 2020. Long-term compound danshen dripping pills therapy reduces the no-reflow phenomenon in nondiabetes mellitus patients after primary percutaneous coronary intervention for acute myocardial infarction. Ann Palliat Med. 9(3):1144–1151. 32498529 10.21037/apm-20-1056

[CIT0002] Bailey DM, Davies B. 2001. Acute mountain sickness; prophylactic benefits of antioxidant vitamin supplementation at high altitude. High Alt Med Biol. 2(1):21–29. 11252695 10.1089/152702901750067882

[CIT0003] Balaban DY, Duffin J, Preiss D, Mardimae A, Vesely A, Slessarev M, Zubieta-Calleja GR, Greene ER, MacLeod DB, Fisher JA. 2013. The *in-vivo* oxyhaemoglobin dissociation curve at sea level and high altitude. Respir Physiol Neurobiol. 186(1):45–52. 23313855 10.1016/j.resp.2012.12.011

[CIT0004] Barshtein G, Ben-Ami R, Yedgar S. 2007. Role of red blood cell flow behavior in hemodynamics and hemostasis. Expert Rev Cardiovasc Ther. 5(4):743–752. 17605652 10.1586/14779072.5.4.743

[CIT0005] Ciaccio C, Di Pierro D, Sbardella D, Tundo GR, Curatolo P, Galasso C, Santarone ME, Casasco M, Cozza P, Cortelazzo A, et al. 2017. Oxygen exchange and energy metabolism in erythrocytes of Rett syndrome and their relationships with respiratory alterations. Mol Cell Biochem. 426(1–2):205–213. 28063007 10.1007/s11010-016-2893-9

[CIT0006] Clarke C. 2006. Acute mountain sickness: medical problems associated with acute and subacute exposure to hypobaric hypoxia. Postgrad Med J. 82(973):748–753. 17099095 10.1136/pgmj.2006.047662 PMC2660503

[CIT0007] Collier DJ, Wolff CB, Hedges A-M, Nathan J, Flower RJ, Milledge JS, Swenson ER. 2016. Benzolamide improves oxygenation and reduces acute mountain sickness during a high-altitude trek and has fewer side effects than acetazolamide at sea level. Pharmacol Res Perspect. 4(3):e00203. 27433337 10.1002/prp2.203 PMC4876137

[CIT0008] Ding F, Yan Y, Huang J, Mei J, Zhu J, Liu H. 2013. The involvement of AQP1 in heart oedema induced by global myocardial ischemia. Cell Biochem Funct. 31(1):60–64. 22865611 10.1002/cbf.2860

[CIT0009] Dong C, Wang G, Li B, Xiao K, Ma Z, Huang H, Wang X, Bai C. 2012. Anti-asthmatic agents alleviate pulmonary edema by upregulating AQP1 and AQP5 expression in the lungs of mice with OVA-induced asthma. Respir Physiol Neurobiol. 181(1):21–28. 22226856 10.1016/j.resp.2011.12.008

[CIT0010] Dosek A, Ohno H, Acs Z, Taylor AW, Radak Z. 2007. High altitude and oxidative stress. Respir Physiol Neurobiol. 158(2–3):128–131. 17482529 10.1016/j.resp.2007.03.013

[CIT0011] Fagenholz PJ, Gutman JA, Murray AF, Harris NS. 2007. Treatment of high altitude pulmonary edema at 4240 m in Nepal. High Alt Med Biol. 8(2):139–146. 17584008 10.1089/ham.2007.3055

[CIT0012] Fan P, Ma H, Jing L, Li L, Jia Z. 2013. The antioxidative effect of a novel free radical scavenger 4'-hydroxyl-2-substituted phenylnitronyl nitroxide in acute high-altitude hypoxia mice. Biol Pharm Bull. 36(6):917–924. 23486089 10.1248/bpb.b12-00854

[CIT0013] Feng P. 2019. Effect of compound Danshen dripping pills on blood oxygen saturation and heart rate of passengers on high altitude trains. Qinghai J Med. 49(04):23–24. Chinese

[CIT0014] Gong G, Yin L, Yuan L, Sui D, Sun Y, Fu H, Chen L, Wang X. 2018. Ganglioside GM1 protects against high altitude cerebral edema in rats by suppressing the oxidative stress and inflammatory response via the PI3K/AKT-Nrf2 pathway. Mol Immunol. 95:91–98. 29428576 10.1016/j.molimm.2018.02.001

[CIT0015] Guilbert C, Chayer B, Allard L, Yu FTH, Cloutier G. 2017. Influence of erythrocyte aggregation on radial migration of platelet-sized spherical particles in shear flow. J Biomechan. 61:26–33. 10.1016/j.jbiomech.2017.06.044 28720200

[CIT0016] Guo J, Yong Y, Aa J, Cao B, Sun R, Yu X, Huang J, Yang N, Yan L, Li X, et al. 2016. Compound danshen dripping pills modulate the perturbed energy metabolism in a rat model of acute myocardial ischemia. Sci Rep. 6:37919. 27905409 10.1038/srep37919 PMC5131350

[CIT0017] Hackett PH, Schoene RB, Winslow RM, Peters RMJ, West JB. 1985. Acetazolamide and exercise in sojourners to 6,300 meters—a preliminary study. Med Sci Sports Exer. 17:593–597. 4068966

[CIT0018] Hall C. 2004. Essential biochemistry and physiology of (NT-pro)BNP. Eur J Heart Fail. 6(3):257–260. 14987573 10.1016/j.ejheart.2003.12.015

[CIT0019] Han J, Li Q, Ma Z, Fan J. 2017. Effects and mechanisms of compound Chinese medicine and major ingredients on microcirculatory dysfunction and organ injury induced by ischemia/reperfusion. Pharmacol Ther. 177:146–173. 28322971 10.1016/j.pharmthera.2017.03.005

[CIT0020] Harrison MF, Anderson PJ, Johnson JB, Richert M, Miller AD, Johnson BD. 2016. Acute mountain sickness symptom severity at the South Pole: the influence of self-selected prophylaxis with acetazolamide. PLoS One. 11(2):e0148206. 26848757 10.1371/journal.pone.0148206 PMC4744068

[CIT0021] Higgins JPT, Green S, editors. 2011. Cochrane handbook for systematic reviews of interventions version 5.1.0 [updated 2011 March]. Res Synth Methods. 2(2):126–130.

[CIT0022] Hsieh S, Tsai J, Yang S, Tang M, Hsieh Y. 2014. Metformin inhibits the invasion of human hepatocellular carcinoma cells and enhances the chemosensitivity to sorafenib through a downregulation of the ERK/JNK-mediated NF-κB-dependent pathway that reduces uPA and MMP-9 expression. Amino Acids. 46(12):2809–2822. 25245054 10.1007/s00726-014-1838-4

[CIT0023] Hu Y, Yagüe E, Zhao J, Wang L, Bai J, Yang Q, Pan T, Zhao H, Liu J, Zhang J. 2018. Sabutoclax, pan-active BCL-2 protein family antagonist, overcomes drug resistance and eliminates cancer stem cells in breast cancer. Cancer Lett. 423:47–59. 29496539 10.1016/j.canlet.2018.02.036

[CIT0024] Imray C, Wright A, Subudhi A, Roach R. 2010. Acute mountain sickness: pathophysiology, prevention, and treatment. Prog Cardiovas Dis. 52(6):467–484. 10.1016/j.pcad.2010.02.003 20417340

[CIT0025] Jacqueline PH, Lorenz R, Inge HUS, M RGMT, M TABM, Daniel B, Otto S, Marco M, R HA. 2010. Changes of coagulation parameters during high altitude expedition. Swiss Med Weekly. 140 (7–8):111–117. 10.4414/smw.2010.12910 19950043

[CIT0026] Jia Q, Zhu R, Tian Y, Chen B, Li R, Li L, Wang L, Che Y, Zhao D, Mo F, et al. 2019. *Salvia miltiorrhiza* in diabetes: a review of its pharmacology, phytochemistry, and safety. Phytomedicine. 58:152871. 30851580 10.1016/j.phymed.2019.152871

[CIT0027] Li K, Li L, Fu J, He Y, Song Z, Zhou S, Gesang L, Liu R. 2020. Efficacy of Danshen (*Salvia miltiorrhiza*) dripping pills for prevention of acute mountain sickness: a randomized, double-blind, placebo-controlled trial. Pakistan J Zool. 52:2215–2226.

[CIT0028] Li L, Weng Z, Yao C, Song Y, Ma T. 2015. Aquaporin-1 deficiency protects against myocardial infarction by reducing both edema and apoptosis in mice. Sci Rep. 5:13807. 26348407 10.1038/srep13807 PMC4562302

[CIT0029] Li Y, Zhang Y, Zhang Y. 2018. Research advances in pathogenesis and prophylactic measures of acute high altitude illness. Respir Med. 145:145–152. 30509704 10.1016/j.rmed.2018.11.004

[CIT0030] Li Z, Gao C, Wang Y, Liu F, Ma L, Deng C, Niu K, Lin M, Wang C. 2011. Reducing pulmonary injury by hyperbaric oxygen preconditioning during simulated high altitude exposure in rats. J Trauma. 71(3):673–679. 21248653 10.1097/TA.0b013e3181f5b073

[CIT0031] Li Z, Guo J, Liu C, Shi Y, Li Y, Wang J, Li D, Wang J, Chen Y. 2020. Compound Danshen dripping pill promotes adaptation to acute high-altitude exposure. High Alt Med Biol. 21(3):258–264. 32466660 10.1089/ham.2019.0126

[CIT0032] Li Z, Xu S, Liu P. 2018. *Salvia miltiorrhiza Burge* (Danshen): a golden herbal medicine in cardiovascular therapeutics. Acta Pharmacol Sin. 39(5):802–824. 29698387 10.1038/aps.2017.193 PMC5943903

[CIT0033] Liang Y, Zou J, Zhang X, Wang Y, Tai J, Guo D, Cui C, Wang J, Cheng J, Shi Y. 2018. The relationship between compound Danshen dripping pills with isosorbide mononitrate in the treatment of elderly patients with unstable angina pectoris. Evid Based Complement Alternat Med. 2018:1–3429151. 10.1155/2018/3429151 PMC6077409 30108652

[CIT0034] Lisk C, McCord J, Bose S, Sullivan T, Loomis Z, Nozik-Grayck E, Schroeder T, Hamilton K, Irwin DC. 2013. Nrf2 activation: a potential strategy for the prevention of acute mountain sickness. Free Radic Biol Med. 63:264–273. 23722164 10.1016/j.freeradbiomed.2013.05.024 PMC4130652

[CIT0035] Liu L, Zhang X. 2017. Effect of compound danshen dropping pill and trimetazidine on blood oxygen saturation and heart rate of medical-team members after entering plateau. Med J Nat Defending Forces Northwest China. 38(04):215–219. Chinese

[CIT0036] Lu W, Zhang X, Cheng S, Shi B. 2016. Protective effects of compound Danshen dripping pills on early diabetic kidney disease and its underlying mechanism. J Xi’an Jiaotong Univ (Med Sci). 37(01):128–133.

[CIT0037] Mairbäurl H, Weber RE. 2012. Oxygen transport by hemoglobin. Comprehen Physiol. 2:1463–1489. 10.1002/cphy.c080113 23798307

[CIT0038] Mannucci PM, Gringeri A, Peyvandi F, Paolantonio TD, Mariani G. 2002. Short-term exposure to high altitude causes coagulation activation and inhibits fibrinolysis. Thromb Haemost. 87(2):342–343. 11858498

[CIT0039] Nieto Estrada VH, Molano Franco D, Medina RD, Gonzalez Garay AG, Martí-Carvajal AJ, Arevalo-Rodriguez I. 2017. Interventions for preventing high altitude illness: part 1. Commonly-used classes of drugs. Cochrane Database Syst Rev. 6(6):CD009761. 28653390 10.1002/14651858.CD009761.pub2 PMC6481751

[CIT0040] Ono Y, Morifusa M, Ikeda S, Kunishige C, Tohma Y. 2017. A case of non-cardiogenic pulmonary edema provoked by intravenous acetazolamide. Acute Med Surg. 4(3):349–352. 29123889 10.1002/ams2.279 PMC5674460

[CIT0041] Pajuelo Reguera D, Čunátová K, Vrbacký M, Pecinová A, Houštěk J, Mráček T, Pecina P. 2020. Cytochrome C oxidase subunit 4 isoform exchange results in modulation of oxygen affinity. Cells. 9(2):443. 10.3390/cells9020443 PMC7072730 32075102

[CIT0042] Pan Y, Zhang Y, Yuan J, Ma X, Zhao Y, Li Y, Li F, Gong X, Zhao J, Tang H, et al. 2020. Tetrahydrocurcumin mitigates acute hypobaric hypoxia-induced cerebral oedema and inflammation through the NF-κB/VEGF/MMP-9 pathway. Phytother Res. 34(11):2963–2977. 32573860 10.1002/ptr.6724

[CIT0043] Reinhart WH, Kayser B, Singh A, Waber U, Oelz O, Bartsch P. 1991. Blood rheology in acute mountain sickness and high-altitude pulmonary edema. J Appl Physiol. 71(3):934–938. 1757331 10.1152/jappl.1991.71.3.934

[CIT0044] Ren J, Fu L, Nile SH, Zhang J, Kai G. 2019. *Salvia miltiorrhiza* in treating cardiovascular diseases: a review on its pharmacological and clinical applications. Front Pharmacol. 10:753. 31338034 10.3389/fphar.2019.00753 PMC6626924

[CIT0045] Roach RC, Hackett PH. 2001. Frontiers of hypoxia research: acute mountain sickness. J Exper Biol. 204(18):3161–3170. 11581330 10.1242/jeb.204.18.3161

[CIT0046] Tan J, Gao C, Wang C, Ma L, Hou X, Liu X, Li Z. 2020. Expression of aquaporin-1 and aquaporin-5 in a rat model of high-altitude pulmonary edema and the effect of hyperbaric oxygen exposure. Dose Response. 18(4):1559325820970821. 33192205 10.1177/1559325820970821 PMC7607770

[CIT0047] Tian K, Tang W, Su Y, Zhang Y, Chen K. 2012. Observation on the effect of treating acute severe altitude sickness in portable pressurized cabin at high altitude. People’s Military Surg. 55:1155–1157. Chinese

[CIT0048] Toff WD, Jones CI, Ford I, Pearse RJ, Watson HG, Watt SJ, Ross JAS, Gradwell DP, Batchelor AJ, Abrams KR, et al. 2006. Effect of hypobaric hypoxia, simulating conditions during long-haul air travel, on coagulation, fibrinolysis, platelet function, and endothelial activation. JAMA. 295(19):2251–2261. 16705106 10.1001/jama.295.19.2251

[CIT0049] Torgovicky R, Azaria B, Grossman A, Eliyahu U, Goldstein L. 2005. Sinus vein thrombosis following exposure to simulated high altitude. Aerospace Med Human Performance. 76:144–146. 15742833

[CIT0050] Tripathi A, Kumar B, Sagi SSK. 2019. Prophylactic efficacy of quercetin in ameliorating the hypoxia induced vascular leakage in lungs of rats. PLOS One. 14(6):e0219075. 31251771 10.1371/journal.pone.0219075 PMC6599121

[CIT0051] Wang C, Jiang H, Duan J, Chen J, Wang Q, Liu X, Wang C. 2018. Exploration of acute phase proteins and inflammatory cytokines in early stage diagnosis of acute mountain sickness. High Alt Med Biol. 19(2):170–177. 29608374 10.1089/ham.2017.0126

[CIT0052] Wang C, Wang R, Xie H, Sun Y, Tao R, Liu W, Li W, Lu H, Jia Z. 2016. Effect of acetazolamide on cytokines in rats exposed to high altitude. Cytokine. 83:110–117. 27104804 10.1016/j.cyto.2016.04.003

[CIT0053] Wei X, Liu Y, Li Q, Yan L, Hu B, Pan C, Li Z, Chang X, Fan J, Zhao N, et al. 2013. Treatment with Cardiotonic pills^®^ after ischemia-reperfusion ameliorates myocardial fibrosis in rats . Microcirculation. 20(1):17–29. 22913380 10.1111/micc.12002

[CIT0054] Wei Y, Lu C, Wei C, Ma Y, Lee H. 2001. Oxidative stress in human aging and mitochondrial disease-consequences of defective mitochondrial respiration and impaired antioxidant enzyme system. Chinese J Physiol. 44(1):1–12. 11403514

[CIT0055] Xiang H, Xue W, Li Y, Zheng J, Ding C, Dou M, Wu X. 2020. Knockdown of ANGPTL2 protects renal tubular epithelial cells against hypoxia/reoxygenation-induced injury via suppressing TLR4/NF-κB signaling pathway and activating Nrf2/HO-1 signaling pathway. Cell Transplant. 29:963689720946663–963689720946663. 32993399 10.1177/0963689720946663 PMC7784569

[CIT0056] Yanamandra U, Boddu R, Pramanik S, Mishra K, Kapoor R, Ahuja A, Chatterjee T, Das S. 2020. Prevalence and clinical characteristics of post-thrombotic syndrome in high-altitude-induced deep vein thrombosis: experience of a single tertiary care center from real-world settings. High Alt Med Biol. 21(4):319–326. 32707006 10.1089/ham.2020.0053

[CIT0057] Yao D, Wang C, Han L, Zhang P, Liu J, Wang B, Zhang E. 2019. Compound danshen dripping pills combined with trimetazidine in treating unstable angina pectoris: protocol for a systematic review of randomized controlled trials. Medicine. 98(49):e18238. 31804349 10.1097/MD.0000000000018238 PMC6919410

[CIT0058] Yao Y, Feng Y, Lin W. 2015. Systematic review and meta-analysis of randomized controlled trials comparing compound danshen dripping pills and isosorbide dinitrate in treating angina pectoris. Int J Cardiol. 182:46–47. 25585358 10.1016/j.ijcard.2014.12.112

[CIT0059] Zhang X, Pei Z, Yan X, Guo Z, Wu D. 2008. Effects of compound danshen dropping pills on cardiovascular response in plateau acclimatization. Chinese J Pathophysiological. 11:2257–2259.

[CIT0060] Zhang X, Zhang X, Dang Z, Su S, Li Z, Lu D. 2020. Cognitive protective mechanism of crocin pretreatment in rat submitted to acute high-altitude hypoxia exposure. Biomed Res Int. 2020:3409679. 32596298 10.1155/2020/3409679 PMC7303745

[CIT0061] Zheng Q, Chen W, Wang Y, Xu X. 2011. The effects of Dan-shen dripping pills on the expression of HIF-1α and VEGF in renal tissues of type 2 diabetic rats. Chinese J Health Care Med. 13:200–203.

[CIT0062] Zhou J, Xun Y, Huang S, Luo Z, Qin Y. 2015. Effect of compound Danshen dripping pills on the function of islet β cells of type 2 diabetic rats. Chinese Trad Patent Med. 37:1807–1810.

