# Supplementary material for: Compound Danshen Dripping Pill inhibits high altitude-induced hypoxic damage by suppressing oxidative stress and inflammatory responses
Source: Pharm Biol. 2021 Nov 22;59(1):1583–91. doi: 10.1080/13880209.2021.1998139 (PMC8635678; doi:10.1080/13880209.2021.1998139)
Supplement: Supplemental Material [file IPHB_A_1998139_SM9910.docx]

Table S1. Hypoxic markers and commercial kits used for their detection in plasma

| Maker name | Catalogue number^a^ |
| --- | --- |
| Intercellular Adhesion Molecule 1 (ICAM1) | H065 |
| Interleukin 1 (IL-1) | H002 |
| Interleukin 6 (IL-6) | H007 |
| Tumor Necrosis Factor α (TNF-α) | H052 |
| NT-proBNP | H334 |
| Matrix Metallopeptidase 9 (MMP9), | H146-4 |
| Total Superoxide Dismutase (TSOD) | A001-1 |
| Glutathione Peroxidase (GPX1) | A005 |

^a^All from Jiancheng Bioengineering Institute, Nanjing, China
